# High Burden of Extended-Spectrum β-Lactamase–Producing *Escherichia coli* and *Klebsiella pneumoniae* Bacteremia in Older Adults: A Seven-Year Study in Two Rural Thai Provinces

**DOI:** 10.4269/ajtmh.18-0394

**Published:** 2019-02-18

**Authors:** Pongpun Sawatwong, Patranuch Sapchookul, Toni Whistler, Christopher J. Gregory, Ornuma Sangwichian, Sirirat Makprasert, Possawat Jorakate, Prasong Srisaengchai, Somsak Thamthitiwat, Chidchanok Promkong, Pongnapat Nanvatthanachod, Muthita Vanaporn, Julia Rhodes

**Affiliations:** 1 Faculty of Tropical Medicine, Mahidol University, Bangkok, Thailand;; 2 Thailand Ministry of Public Health (MOPH)-U.S. Centers for Disease Control and Prevention Collaboration (TUC), Nonthaburi, Thailand;; 3 Division of Global Health Protection, Centers for Disease Control and Prevention, Atlanta, Georgia;; 4 Nakhon Phanom Provincial Hospital, Nakhon Phanom, Thailand;; 5 Sa Kaeo Crown Prince Hospital, Sa Kaeo, Thailand

## Abstract

Bloodstream infection surveillance conducted from 2008 to 2014 in all 20 hospitals in Sa Kaeo and Nakhon Phanom provinces, Thailand, allowed us to look at disease burden, antibiotic susceptibilities, and recurrent infections caused by extended-spectrum β-lactamase (ESBL)–producing *Escherichia coli* and *Klebsiella pneumoniae*. Of 97,832 blood specimens, 3,338 were positive for *E. coli* and 1,086 for *K. pneumoniae*. The proportion of *E. coli* isolates producing ESBL significantly increased from 19% to 22% in 2008–2010 to approximately 30% from 2011 to 2014 (*P*-value for trend = 0.02), whereas ESBL production among *K. pneumoniae* cases was 27.4% with no significant trend over time. Incidence of community-onset ESBL-producing *E. coli* increased from 5.4 per 100,000 population in 2008 to 12.8 in 2014, with the highest rates among persons aged ≥ 70 years at 79 cases per 100,000 persons in 2014. From 2008 to 2014, community-onset ESBL-producing *K. pneumoniae* incidence was 2.7 per 100,000, with a rate of 12.9 among those aged ≥ 70 years. Although most (93.6% of *E. coli* and 87.6% of *K. pneumoniae*) infections were community-onset, hospital-onset infections were twice as likely to be ESBL. Population-based surveillance, as described, is vital to accurately monitor emergence and trends in antimicrobial resistance, and in guiding the development of rational antimicrobial therapy recommendations.

## INTRODUCTION

*Escherichia coli* and *Klebsiella pneumoniae* are two of the most common causes of bloodstream infections (BSIs) worldwide.^1,2^ β-Lactam antibiotics are the preferred treatment because of their selective toxicity, broad spectrum of activity, and low cost.^3,4^ However, *E. coli* and *K. pneumoniae*, and other Enterobacteriaceae, have developed extended-spectrum β-lactamase (ESBL)–producing capability and can hydrolyze penicillins, cephalosporins, and monobactams, rendering them clinically ineffective.^5^ Extended-spectrum β-lactamase–producing *E. coli* and *K. pneumoniae* are increasingly reported worldwide in hospital and community settings, resulting in prolonged and more expensive hospitalizations and increased morbidity and mortality.^6,7^ Prompt identification is essential for appropriate treatment and improved patient outcomes.^8,9^

Extended-spectrum β-lactamase–producing *E. coli* have been identified throughout Thailand,^10–13^ with the first documented case in 1994,^14^ and a prevalence among different clinical specimens ranging from 22% to 59%, and evidence that they are becoming more common.^10–13,15^ However, representative estimates of the incidence of ESBL-producing *E. coli* and *K. pneumoniae* and longitudinal trends in antimicrobial resistance are currently limited in Southeast Asia.

This study aimed to expand our understanding of ESBL-producing pathogen disease burden by estimating trends in population-based *E. coli* and *K. pneumoniae* bacteremia incidence, changes over time in the proportion of these cases due to ESBL-producing organisms, susceptibility profiles to other antibiotics, and the probability of recurrent infections. This study used the same well-established surveillance database that was previously used to estimate the incidence and susceptibility profiles of several other pathogens in rural Thailand.^16–19^

## MATERIAL AND METHODS

### Study setting.

We conducted comprehensive BSI surveillance in two rural provinces: Sa Kaeo (SK) in eastern Thailand bordering Cambodia and Nakhon Phanom (NP) in northeast Thailand bordering Laos People’s Democratic Republic. Both provinces are agrarian with estimated populations of approximately 526,000 and 734,000, respectively, from National Economic and Social Development Board (NESDB) projections based on 2010 census data.^20,21^ This study included data between 2008 and 2014 from all 20 hospitals of the two provinces (16 district, two military [10–140 beds], and two provincial hospitals [225–327 beds]), and included all laboratories performing hemoculture.^22^

### Specimen collection and laboratory testing.

As clinically indicated, hemocultures were performed using two different conditions, one optimized for aerobic growth and the other for enhanced growth of mycobacteria and other fastidious organisms as previously reported. A modification was applied in October 2011; the mycobacterial type bottles were not routinely processed but available on physician request.^23^ Hemoculture bottles inoculated at district or military hospitals were kept at 15–30°C and transported to the provincial laboratory within 24 hours before placement in the BacT/ALERT 3D system (BioMérieux, Marcy-l’Étoile, France). Alarm-positive bottles were subcultured on chocolate, sheep blood, and MacConkey agars and incubated overnight at 35°C. Standard biochemical tests were performed for organism identification.^24^ From 2012, Analytical Profile Index Microbial Identification Kits (BioMérieux) were used when standard testing was not definitive. *Escherichia coli*- and *K. pneumoniae*-positive cultures that grew a likely contaminant in the same bottle were excluded from our case counts. Repeat positive cultures that grew the same species within 30 days were excluded.

Antibiotic susceptibility testing was performed using a modified Kirby–Bauer disk diffusion method.^25^ To improve screen test sensitivity, ESBL screening used two single-disc diffusion tests: ceftazidime (CAZ) and cefotaxime (CTX) with zones of inhibition of ≤ 22 or ≤ 27 mm, respectively, were considered positive.^25^ Confirmatory testing used a combination disc method. Ceftazidime/Clavulanic acid (CAZ/CLA) or CTX/CLA discs were placed on the same culture plates as the single discs. A ≥ 5-mm increase in combination disc zone diameter compared with single disc alone confirmed ESBL production. Confirmation testing was routinely performed in SK. Nakhon Phanom started confirmatory testing in 2014. We defined an ESBL-producing pathogen as an isolate that was either confirmation or screen test positive when confirmatory testing was not available.

Multiplex polymerase chain reaction for five common genetic mechanisms of resistance (*bla*_NDM-1_, *bla*_OXA_, *bla*_IMP_, *bla*_KPC_, and *bla*_VIM_) were performed as previously described.^26^

### Definitions and data analysis.

Likely contaminants that grew in the same hemoculture bottle (51 *E. coli*- and 20 *K. pneumoniae*-positive cultures) were excluded from case counts. These were *Staphylococcus* spp. other than *Staphylococcus aureus* (*n* = 28), Gram-positive bacillus (*n* = 16), *Bacillus* spp. other than *Bacillus anthracis* (*n* = 15), *Streptococcus viridans* group (*n* = 8), and other (*n* = 4).

*Escherichia coli* and *K. pneumoniae* cases were classified as hospital onset (HO) when the hemoculture was drawn after day 3 of hospital stay, with day of admission being day 1.^27^ Community onset (CO) cases were described by a positive hemoculture collected on or before day 3 of hospital stay. Preculture antibiotic use was any antibiotic received within 72 hours of culture determined by patient report and review of medical records. Recurrent infections were defined as positive hemoculture for the same pathogen more than 30 days after the initial positive culture. Mortality outcome was based on information available at the time of hospital discharge and, therefore, reflects solely in-hospital mortality.

The CO incidence rate of *E. coli* and *K. pneumoniae* bacteremia was calculated using age-specific population estimates from the NESDB.^20^ For the period 2007–2009, the 2010 NESDB age distribution was applied to the 2007–2009 NESDB overall provincial population estimates,^20^ as official intercensal estimates were not available.^21^ To calculate HO incidence cases, we used the number of hospitalizations from 2008 to 2014 from hospital registration databases (S. Naorat and B. Piralam, personal communication). In SK, four district hospitals were missing 2008 and 2009 data. Together, these hospitals account for ≤ 15% of annual hospitalizations in our surveillance system, so we imputed hospitalization data from 2010 data for these four hospitals. Military hospitals were also excluded from HO incidence calculations, as hospitalization data were not available. Military hospitals account for < 1% of hospitalizations in each year of surveillance.

The chi-squared or Fisher exact tests were used as appropriate. Ninety-five percent CIs on incidence estimates were calculated from the normal approximation to the Poisson distribution.

### Ethical considerations.

The CDC Human Subjects Review Office determined that this protocol constituted routine public health activities and did not involve human subject research (CGH 2014-273).

## RESULTS

### Blood culturing practices and bacteremia incidence.

Between 2008 and 2014, 97,832 hemocultures were performed, with 12,813 (13.1%) positive for a pathogen. This excludes 3,859 cultures that grew a likely contaminant and pathogen in the same culture bottle. A total of 3,338 *E. coli* infections (1,595 from SK and 1,743 from NP) and 1,086 *K. pneumoniae* infections (529 from SK and 557 from NP) were identified. Annual *E. coli* bacteremia case counts increased from 373 in 2008 to 593 in 2014, with corresponding increases in annual incidence from 32.9 (2008) to 51.6 (2014) per 100,000 persons. Likewise, *K. pneumoniae* case counts increased from 116 in 2008 to 178 in 2014, with corresponding increases in incidence from 10.2 (2008) to 15.5 (2014) per 100,000 persons.

### Extended-spectrum β-lactamase screening results.

**Figure 1.**
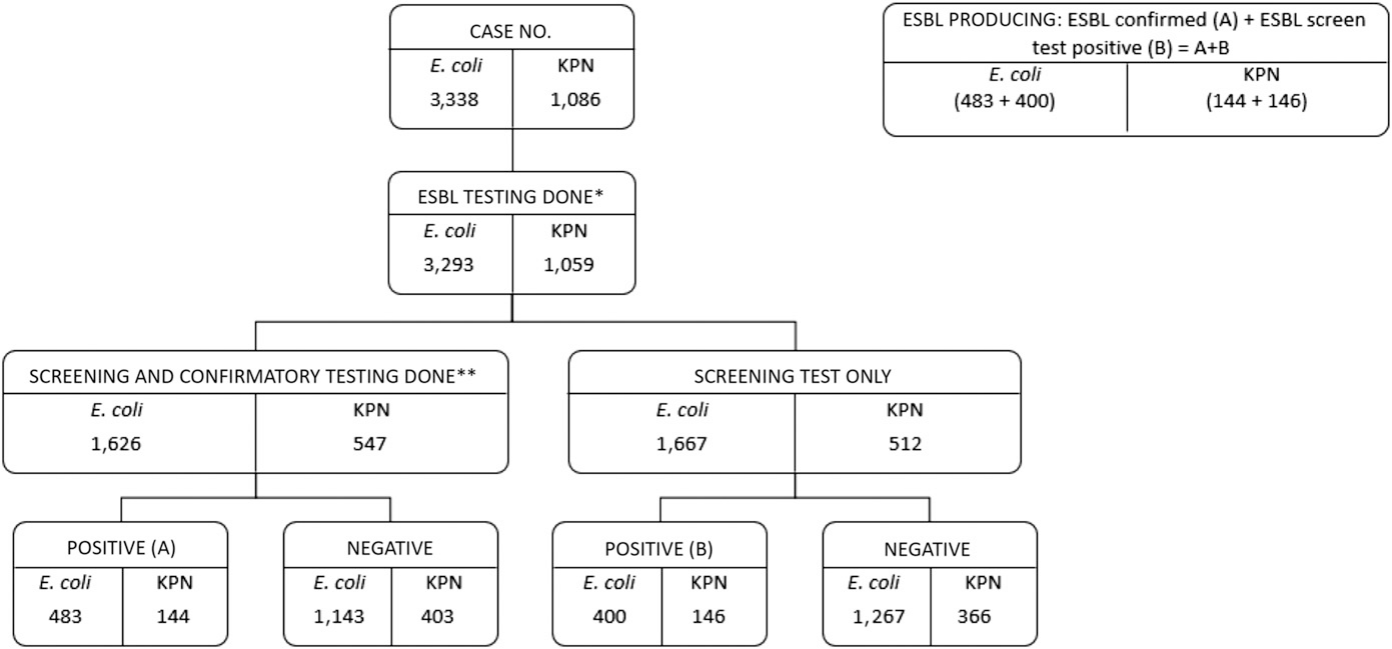
Extended-spectrum β-lactamase (ESBL)–producing *Escherichia coli* and *Klebsiella pneumoniae* (KPN) bacteremia cases in hospitalized patients from Nakhon Phanom and Sa Kaeo provinces, Thailand, 2008–2014. *Screening was performed using a single-disc diffusion test and a positive test was defined as a zone of inhibition for ceftazidime (CAZ) ≤ 22 mm OR cefotaxime (CTX) ≤ 27 mm. **Confirmatory testing was a combination disc method. The organism was confirmed as an ESBL producer if there was an increase of ≥ 5 mm in the zone diameter of CAZ/CLA disc compared with CAZ disc alone, or ≥ 5 mm increase in the zone diameter of CTX/CLA disc compared with a CTX disc alone. Confirmatory testing was not available in Nakhon Phanom Province before 2014. For *E. coli*, 85.3 % (1,387/1,626) and 83.9% (459/547) of *K. pneumoniae* isolates with confirmatory testing were from Sa Kaeo.

Extended-spectrum β-lactamase screening was performed for 98.7% (3,293/3,338) of *E. coli* and 97.5% (1,059/1,086) of *K. pneumoniae* isolates (Figure 1). Comparisons of single-disc screening versus combination disc confirmatory tests demonstrated that screen tests had a high sensitivity (98.4% in *E. coli* and 95.7% in *K. pneumoniae*), specificity (93.8% in *E. coli* and 92.1% in *K. pneumoniae*), and overall agreement (95.5% [1,324/1,387] for *E. coli* and 93.0% [427/459] for *K. pneumoniae*). We, therefore, included screen test only positive isolates with confirmatory test positives as “ESBL producing.” Overall, 26.8% (883/3,293) of *E. coli* and 27.4% (290/1,059) of *K. pneumoniae* isolates were ESBL producing (Figure 1).

### Overall proportion and trend of ESBL-producing *E. coli* and *K. pneumoniae* bacteremia.

Extended-spectrum β-lactamase–producing capability among *E. coli* bacteremia cases ranged from 23.8% to 31.9% by age group; however, ESBL-producing *K. pneumoniae* was significantly more common among children aged 0–4 years compared with older persons (*P* < 0.01, Table 1). Prevalence of ESBL in *E. coli* increased over time: from 19% to 22% in 2008–2010 to approximately 30% from 2011 to 2014 (*P*-value for trend = 0.02). Prevalence of ESBL among *K. pneumoniae* cases had no apparent trend over time (*P* = 0.09). There were no statistically significant differences between the two provinces for the percentage of *E. coli* and *K. pneumoniae* that were ESBL producing (Table 1).

**Table 1:** Characteristics of patients with *Escherichia coli* and *Klebsiella pneumoniae* bacteremia, Nakhon Phanom and Sa Kaeo provinces, Thailand, 2008–2014

Demographic data	*E. coli*, *N* = 3,293, n* (%)			*K. pneumoniae*, *N* = 1,059, n* (%)		
	ESBL, *N* = 883	Non-ESBL, *N* = 2,410	*P*-value	ESBL, *N* = 290	Non-ESBL, *N* = 769	*P*-value
Province						
Nakhon Phanom	436 (25.4)	1,281 (74.6)	0.06	157 (29.1)	382 (70.9)	0.22
Sa Kaeo	447 (28.4)	1,129 (71.6)	–	133 (25.6)	387 (74.4)	–
Age group (years)						
Median age (interquartile range)	62 (52–73)	63 (51–74)	–	57 (43–71)	62 (50–73)	–
0–4	23 (31.9)	49 (68.1)	0.26	27 (58.7)	19 (41.3)	< 0.01
5–19	10 (23.8)	32 (76.2)	1.00	6 (46.2)	7 (53.8)	0.16
20–49	159 (25.2)	471 (74.8)	Ref	63 (28.1)	161 (71.9)	Ref
50–69	408 (29.2)	991 (70.8)	0.07	118 (27.2)	316 (72.8)	0.85
70+	282 (24.6)	866 (75.4)	0.77	75 (22.0)	266 (78.0)	0.11
Year enrolled†						
2008	73 (19.6)	300 (80.4)	Ref	30 (25.9)	86 (74.1)	Ref
2009	109 (23.2)	360 (76.8)	0.21	32 (24.6)	98 (75.4)	0.88
2010	99 (22.0)	351 (78.0)	0.44	44 (27.7)	115 (72.3)	0.78
2011	120 (29.8)	283 (70.2)	< 0.01	38 (31.1)	84 (68.9)	0.39
2012	143 (29.3)	345 (70.7)	< 0.01	58 (32.8)	119 (67.2)	0.24
2013	169 (32.7)	348 (67.3)	< 0.01	43 (24.3)	134 (75.7)	0.78
2014	170 (28.7)	423 (71.3)	< 0.01	45 (25.3)	133 (74.7)	1.00
Onset						
Community	777 (25.2)	2,306 (74.8)	< 0.01	219 (23.6)	709 (76.4)	< 0.01
Hospital	106 (51.7)	99 (48.3)	–	71 (55.9)	56 (44.1)	–
Prior hospitalization (past 365 days)						
Yes	65 (63.7)	37 (36.3)	< 0.01	48 (71.6)	19 (28.4)	< 0.01
No	818 (25.6)	2,373 (74.4)	–	242 (24.4)	750 (75.6)	–
Hospital						
Provincial	420 (27.1)	1,131 (72.9)	0.37	181 (30.6)	410 (69.4)	0.01
District	404 (25.7)	1,170 (74.3)	–	93 (22.6)	319 (77.4)	–

ESBL = extended-spectrum β-lactamase.

* *n* varies slightly because of data availability.

† *E. coli* trend over time, *P*-value = 0.018; *K. pneumoniae* trend over time, *P*-value = 0.103.

### Clinical features and risk factors for infection with ESBL-producing *E. coli* and *K. pneumoniae*

Persons aged ≥ 50 years accounted for 77.3% (2,547/3,293) of *E. coli* bacteremia cases and 78.1% (690/883) of ESBL-producing *E. coli* cases. Similarly, 73.2% (775/1,059) of *K. pneumoniae* bacteremia cases and 66.6% (193/290) of ESBL-producing *K. pneumoniae* cases were in persons aged ≥ 50 years. Among children aged < 5 years, neonates ≤ 28 days accounted for 50.0% (36/72) of *E. coli* bacteremia, 60.9% (14/23) of ESBL-producing *E. coli*, 58.7% (27/46) of *K. pneumoniae* cases, and 63.0% (17/27) ESBL-producing *K. pneumoniae*.

The majority of *E. coli* (93.6%) and *K. pneumoniae* cases (87.6%) were CO; however, HO cases were twice as likely to be ESBL-producing compared with CO cases. For *E. coli*, 51.7% (106/205) of HO cases were identified as ESBL versus 25.2% (777/3,083) of the CO cases (*P* < 0.01). For *K. pneumoniae*, 55.9% (71/127) of HO cases were ESBL versus 23.6% (219/928) of CO cases (*P* < 0.01) (Table 1).

Hospitalization in the past year was significantly more frequent among patients with ESBL-producing *E. coli* and *K. pneumoniae* (63.7% and 71.6%, respectively) than in patients with non-ESBL–producing organisms (36.3% and 28.4%, respectively). The percentage of ESBL *E. coli* did not differ between provincial and district level hospitals (27.1% versus 25.7%, *P* = 0.37), but ESBL *K. pneumoniae* was significantly more common in provincial level hospitals than district hospitals (30.6% versus 22.6%, *P* = 0.01).

Among those with available outcome data, the mortality rate was twice as high among case patients with ESBL-producing *E. coli* (14.5%, 56/387) versus 7.1% (85/1,202) for non-ESBL–producing *E. coli* (*P* < 0.001), and this difference persisted after age stratification (*P* < 0.01). In fact, mortality was nearly three times higher among patients aged 70 years and older with ESBL-producing *E. coli* (20.0%, 26/130) than among patients with non-ESBL–producing *E. coli* (7.4%, 33/446). Among children aged 0–19 years, there were no deaths among 35 patients with non-ESBL *E. coli* compared with two deaths among 13 patients (15.4%) with ESBL-producing *E. coli*. But for *K. pneumoniae* case patients, the mortality rate was not different between those with ESBL-producing and non-ESBL–producing infections (19.1% [25/131] and 14.4% [56/390], respectively) (*P* = 0.197).

### Trends in community-onset ESBL-producing *E. coli* and *K. pneumoniae* incidence.

Overall CO ESBL-producing *E. coli* bacteremia incidence during 2008–2014 was 9.8 cases per 100,000 persons per year (95% CI: 9.1–10.5). This incidence increased from 5.4 (95% CI: 4.1–6.7) in 2008 to 12.8 per 100,000 persons per year (95% CI: 10.7–14.9) in 2014. The community onset ESBL-producing *K. pneumoniae* bacteremia incidence from 2008 to 2014 was 2.7 per 100,000 (95% CI: 2.4–3.1), from a low of 2.2 (95% CI: 1.3–3.0) in 2008 to a high of 4.0 (95% CI: 2.8–5.1) in 2012.

**Figure 2.**
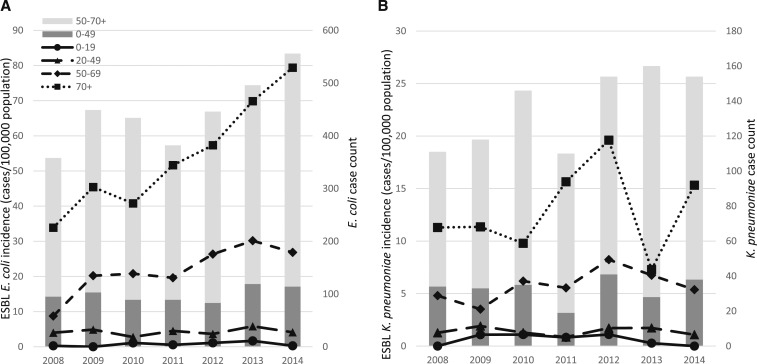
Community onset incidence of extended-spectrum β-lactamase (ESBL)–producing *Escherichia coli* (**A**) and *Klebsiella pneumoniae* (**B**) bacteremia by age group, 2008–2014.

The estimated incidence of CO ESBL-producing *E. coli* was highest among persons aged ≥ 70 years, and this age group showed the most pronounced increase from 34 cases/100,000 population in 2008 (95% CI: 19–48) to 79 cases/100,000 population (95% CI: 59–100) in 2014 (Figure 2). Incidence among persons aged 50–69 years also increased over time from 9/100,000 (95% CI: 5–13) in 2008 to 26–30/100,000 (95% CI: 20–37) in 2012–2014. Incidence was lower among persons aged 20–49 and 0–19 years and remained stable over time. The overall estimate for these age groups during the 2008–2014 period was 4.2/100,000 (95% CI: 3.5–4.9) and 0.7/100,000 (95% CI: 0.4–1.0), respectively. This pattern is consistent with the increase in overall *E. coli* case counts during this period which was primarily limited to patients older than 50 years (Figure 2). Similarly, CO ESBL-producing *K. pneumoniae* incidence was highest in persons aged ≥ 70 years (12.9 cases/100,000 [95% CI: 9.6–16.3]), followed by persons aged 50–69 years (5.6 cases/100,000 [95% CI: 4.4–6.7]) and persons aged 20–49 years at 1.4 cases/100,000 (95% CI: 0.2–2.3), and lowest in persons aged 0–19 years at 0.6 cases/100,000 (95% CI: 0.3–0.9) (Figure 2). However, unlike *E. coli*, CO ESBL-producing *K. pneumoniae* incidence did not increase significantly in any of these age categories over time (Figure 2). The number of cases of ESBL-producing *K. pneumoniae* remained stable despite an increase in total *K. pneumoniae* case counts over time. Higher *K. pneumoniae* case counts in 2013–2014 were offset by a lower percentage of ESBL-producing infections (25%) compared with that in 2011–2012 (31–32%).

### Trends in hospital-onset ESBL-producing *E. coli* and *K. pneumoniae* incidence.

The incidence of HO ESBL-producing *E. coli* during the 7-year period varied between 0.13 and 0.30 cases/1,000 hospitalizations in SK. This was significantly higher than that in NP, where the incidence was 0.06 cases/1,000 hospitalizations in 2008, which dropped to 0.01 in 2009 and increased to 0.12 cases per 1,000 hospitalizations in 2014 (*P* < 0.01 for the trend between 2009 and 2014). Initially, the incidence of HO ESBL-producing *K. pneumoniae* from 2008 to 2014 was higher in SK at 0.12 (95% CI: 0.09–0.16) cases/1,000 population compared with 0.05 cases/1,000 population (95% CI: 0.03–0.07) in NP (*P* = 0.03). However, HO *K. pneumoniae* bacteremia incidence increased significantly over time in NP (*P* = 0.03) and by 2014 estimates from the two sites were similar; in 2014, HO ESBL-producing *K. pneumoniae* was 0.11 (95% CI: 0.0–0.2) in NP and 0.13 (95% CI: 0.0–0.2) in SK.

### Antimicrobial usage and microbiological characteristics of ESBL and non-ESBL–producing infections.

Most *E. coli* (88.8%; 2,500/2,815) and *K. pneumoniae* (86.0%; 772/898) case patients did not receive antibiotics in the 72 hours before blood draw; however, pre-culture antibiotic use was three to four times more common among ESBL-producing case patients than in non-ESBL–producing case patients (Table 2). The most common pre-culture antibiotics used were cephalosporins, which was significantly higher among ESBL-producing cases (Table 2).

**Table 2:** Antibiotic use before blood draw among hospitalized bacteremia cases with extended-spectrum β-lactamase (ESBL) and non-ESBL production for *Escherichia coli* and *Klebsiella pneumoniae*, Nakhon Phanom and Sa Kaeo provinces, Thailand, 2008–2014

Antibiotic use before blood draw	*E. coli*					*K. pneumoniae*				
	ESBL, *N* = 746		Non-ESBL, *N* = 2,069		*P*-value*	ESBL, *N* = 237		Non-ESBL, *N* = 661		*P*-value*
	*n*	%	*N*	%		*n*	%	*n*	%	
No preculture antibiotic use	553	74.1	1,947	94.1	Ref	164	69.2	608	92.0	Ref
Preculture antibiotic use	193	25.9	122	5.9	< 0.01	73	30.8	53	8.0	< 0.01
β-Lactam (narrow spectrum)	7	3.6	5	4.1	< 0.01	4	5.5	6	11.3	0.15
β-Lactam (extended spectrum)	13	6.7	19	15.6	0.02	12	16.4	11	20.8	< 0.01
Cephalosporins	138	71.5	39	32.0	< 0.01	42	57.5	9	17.0	< 0.01
Aminoglycosides	7	3.6	8	6.6	0.02	10	13.7	3	5.7	< 0.01
Carbapenems	3	1.6	1	0.8	0.01	0	0	1	1.9	0.06
Fluoroquinolones	19	9.8	17	13.9	< 0.01	11	15.1	5	9.4	< 0.01
Macrolides	17	8.8	9	7.4	< 0.01	6	8.2	5	9.4	0.01
Nitromidazole	29	15.0	13	10.7	< 0.01	6	8.2	2	3.8	< 0.01
Combination (received > 1 antibiotic)	67	34.7	23	18.9	< 0.01	7	9.6	31	58.5	< 0.01
Others†	11	5.7	22	18.0	0.14	7	9.6	12	22.6	0.1

* *P*-values on chi-squared tests comparing no pre-culture antibiotic use vs each class of antibiotics.

† Others: Amphotericin, sulperazone, augmentin, bactrim, and cotrimoxazole.

**Figure 3.**
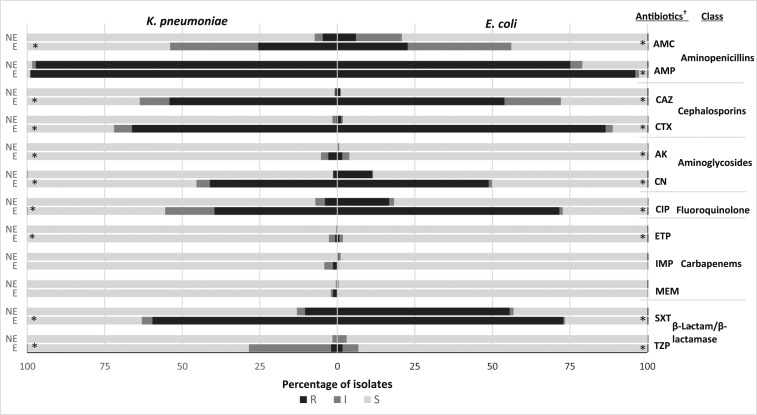
Antibiotic susceptibility in community onset patients of *Escherichia coli* and *Klebsiella pneumoniae*, 2008–2014. *Antibiotic discs used were ampicillin (AMP, 10 µg), ceftazidime (CAZ, 30 µg), gentamicin (CN, 10 µg), amikacin (AK, 30 µg), amoxicillin (AMC, 10 µg), piperacillin/tazobactam (TZP, 100/10 µg), cefotaxime (CTX, 30 µg), ciprofloxacin (CIP, 5 µg), ertapenem (ETP, 10 µg), imipenem (IMP, 10 µg), meropenem (MEM, 10 µg), and trimethoprim–sulfamethoxazole (SXT, 1.25/23.75 µg). †*P*-value < 0.05. E = ESBL producing; NE = non-ESBL producing.

There were significantly different antibiotic susceptibility profiles between ESBL and non-ESBL CO *E. coli* infections. Extended-spectrum β-lactamase CO *E. coli* infections had significantly lower susceptibility to amoxicillin (43.9% versus 79.2%), ceftazidime (27.9% versus 98.9%), cefotaxime (11.2% versus 98.3%), gentamycin (50.1% versus 88.5%), ciprofloxacin (27.3% versus 81.8%), and trimethoprim/sulfamethoxazole (26.6% versus 43.2%) (Figure 3). Similarly for *K. pneumoniae* CO infections, there was lower susceptibility for amoxicillin (46.2% versus 92.7%), ceftazidime (36.4% versus 99.1%), cefotaxime (28.0% versus 98.4%), gentamycin (54.6% versus 98.5%), ciprofloxacin (44.4% versus 92.9%), and trimethoprim/sulfamethoxazole (37.0% versus 87.0%) (Figure 3).

Among HO cases, a significant difference in susceptibility was seen between ESBL and non-ESBL–producing *E. coli* for third-generation cephalosporins (4.8% versus 94.9% for cefotaxime and 12.5% versus 95.9% for ceftazidime), fluoroquinolone (32.4% versus 78.4% in ciprofloxacin), and aminopenicillins (34.6% versus 77.6% in amoxicillin) (all *P* < 0.001) (but not for aminoglycosides, carbapenems, and antibiotic combinations involving a β-lactamase inhibitor). More than 85% of HO ESBL-producing *E. coli* and *K. pneumoniae* were susceptible to piperacillin/tazobactam, amikacin, and carbapenems. Similar differences in susceptibility to cephalosporins, fluoroquinolones, and aminopenicillins were seen among HO *K. pneumoniae* cases.

Of 864 ESBL-producing *E. coli*, two were carbapenem-resistant Enterobacteriaceae (CRE) (one in 2012 encoding genes other than five tested for, and one in 2014 carrying *bla*_NDM-1_ gene). Of 277 ESBL-producing *K. pneumoniae*, three were CRE (two cases with *bla*_NDM-1_ in 2013 and 2014, and one case in 2012 encoding genes not tested for). Of 2,400 non-ESBL–producing *E. coli*, two were CRE (one case in 2011 encoding *bla*_IMP_ and another in 2014 encoding genes not covered). There were no non-ESBL–producing *K. pneumoniae* CRE cases.

### Recurrent *E. coli* and *K. pneumoniae* bacteremias.

**Figure 4.**
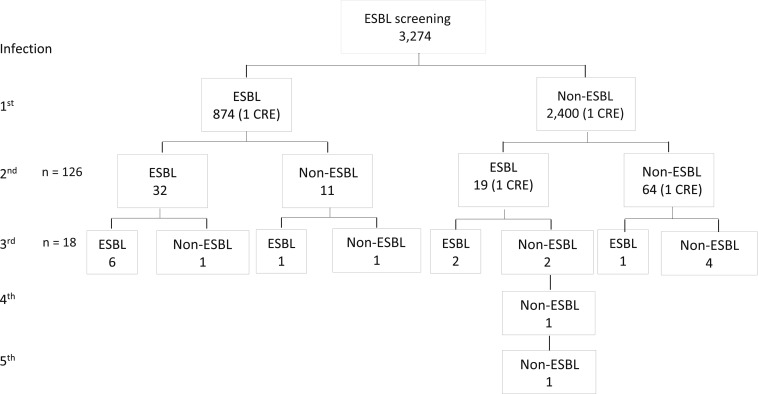
Recurrent *Escherichia coli* infections. Recurrent infections were defined as positive blood cultures for the same pathogen more than 30 days after the initial positive blood culture.

Recurrent bacteremia was noted in 3.8% (126/3,274) of *E. coli* case patients. These 126 patients had a total of 271 episodes; 14.3% (18/126) had ≥ 3 episodes of *E. coli* bacteremia. Of the 271 bacteremia cases, 105 (38.7%) were ESBL, which was similar to the overall percent of ESBL among *E. coli* infections. At least a quarter (25.9% or 14/54) of the recurrent bacteremia was new, as a non-ESBL infection was preceded by an ESBL infection (Figure 4). Recurrent bacteremia was present in 2.7% (28/1,043) of patients with *K. pneumoniae* and 28.6% (8/28) were ESBL-producing, similar to the overall percentage among *K. pneumoniae*.

## DISCUSSION

This is one of the largest population-based studies to estimate the incidence of ESBL-producing organisms. It includes almost 100,000 specimens, ordered for clinical purposes from all hospitals in two rural Thai provinces over a 7-year period, providing large-scale, longitudinal antibiotic usage and resistance data, and documenting increasing incidence of ESBL production and the development of CRE in these areas.

Our study shows more than 25 percent of *E. coli* and *K. pneumoniae* isolates causing bacteremia in hospitalized patients from 2008 to 2014 were ESBL producers. The proportion of these isolates increased over time leading to a notable upsurge in the incidence of ESBL *E. coli* bacteremia, particularly among persons ≥ 50 years old. Our findings agree with increases in ESBL incidence and other antibiotic resistance in *E. coli* over the past 15–20 years seen in such diverse locations as Hong Kong,^28^ Canada,^29^ and Malawi.^30^ However, this was not a universal finding as shown by stable levels of resistance in New Zealand from 2005 to 2011.^31^ Variations in overall incidence and trends in antimicrobial resistance are likely driven in part by antibiotic usage patterns in different locations. In our study, patients with ESBL-producing infections were more likely to have received antibiotics, most frequently cephalosporins, in the preceding 72 hours similar to that previously documented in a Korean study.^32^ It is difficult to discern whether cephalosporin use led to the development of ESBL production or whether it resulted in clearance of non-ESBL–producing infections. Extended-spectrum β-lactamase-producing infections were associated with increased mortality in our study, which is consistent with previous reports from tertiary-level hospitals in Thailand^10^ and elsewhere.^6,7^

Our findings that ESBL-producing *E. coli* and *K. pneumoniae* are causing both CO and HO bacteremia agree with other studies in Thailand,^33,34^ and the Thai studies that looked at temporal trends found ESBL-producing *E. coli* isolates are increasing.^34^

Two factors explain the increase in incidence: a growing total case count and an increasing percentage of *E. coli* infections caused by ESBL-producing organisms. As in other studies, the increase in ESBL *E. coli* infections appears to add to the total burden of BSIs rather than represent a replacement of ESBL-negative with ESBL-positive infections.^28^ Our observed prevalence of ESBL-producing capability among *E. coli* (22.0%) and *K. pneumoniae* (27.7%) isolates are higher than 2010 estimates from Kanoksil et al.^34^ They reported an increase in the percentage of CO bacteremia due to ESBL-producing *E. coli* and *K. pneumoniae* in Northeast Thailand between 2004 and 2010 (2.9–18.0% and 10.0–16.4%, respectively). Part of this difference is likely due to variations in antibiotic susceptibility testing methodologies between the multiple provincial hospitals included in the Kanoksil study.

There are very few studies which provide population-based estimates of the incidence of ESBL-producing *E. coli* and *K. pneumoniae* globally. In Canada, an overall incidence of CO infections from ESBL-producing *E. coli* from all specimens of 7.7–13.3 per 100,000 population between 2004 and 2006 was reported, with blood accounting for only 10% of specimens. In our study, the incidence of CO ESBL-producing *E. coli* BSIs alone was 5.4–13.3 per 100,000 population. This demonstrates an incidence of ESBL-producing *E. coli* BSIs 8–10 times higher than in Canada.^35^ Despite this, the age-related patterns of infection were similar with the highest incidence in persons aged > 70 years and an increasing incidence in people aged older than 50 years. Similar age patterns have been seen in multiple geographic locations for overall *E. coli* and all etiology BSI incidence.^30,31,36^

According to projections for Thailand 2010–2040 by NESDB,^20^ the population of persons over 50 years old is rising rapidly. If disease incidence remained at 2014 levels, by the year 2020, the number of CO ESBL-producing *E. coli* bacteremia cases will increase 19% in NP and SK because of the aging population alone. If we factor in the increasing incidence over time (1.2 cases/year), by 2020 case counts will increase 63% compared with that in 2014. Community onset ESBL-producing *K. pneumoniae* bacteremia incidence was stable, but application of the 2008–2014 *K. pneumoniae* incidence estimate to the projected 2020 population age structure results in an estimated 20% increase in cases compared with 2014.

About 3.8 percent of *E. coli* bacteremia were recurrent infections. This has been shown in other population-based studies to be strongly associated with HO infections and an independent predictor for death.^37^ Our recurrence rate was slightly higher than the 2.4% documented in a 7-year population-based Canadian study.^29^

The antibiotic treatment choices for infections caused by ESBL-producing pathogens are limited. We found a high prevalence of resistance to third-generation cephalosporins among ESBL-producing *E. coli* and *K. pneumoniae*, which is consistent with previous reports from Thailand.^38^ A 2016 report^39^ examined treatment options for children (0–15 years) with *E. coli* and *K. pneumonia*e infections visiting a tertiary care center in Bangkok. They also found the vast majority of ESBL-producing isolates were susceptible to amikacin but noted that poor tissue penetration and potential nephrotoxicity limited the use of amikacin and other aminoglycosides outside of urinary tract infections.^40^ Another potential treatment option is piperacillin/tazobactam, but we found substantial non-susceptibility (> 25%) to this combination among ESBL-producing *K. pneumoniae* isolates, similar to Sethaphanich,^39^ who found only 62.6% of *K. pneumoniae* isolates were susceptible to piperacillin/tazobactam. This makes it a poor choice for empiric treatment. For now, carbapenems remain a reliable treatment option, but it is troubling that we identified carbapenem-resistant, ESBL-producing isolates from five patients (two *E. coli* and three *K. pneumoniae*), especially in light of the recent identification of colistin-resistant *E. coli* from clinical isolates, healthy adults, and the environment in Thailand.^41,42^ If we cannot prevent and control the spread of these types of multidrug-resistant bacteria, few options will remain for patients infected with this organism in the near future.

Our study had several limitations, including missing data to determine whether CO infections were health care associated or not. The retrospective nature and limited scope of data collection did not allow us to identify risk factors (apart from recent antibiotic use) for the presence of an ESBL-producing infection or potential primary foci of infection. Outcome data were only available for the period of hospital admission, which likely led to an underestimated case fatality rate, as previous work in rural Thailand show that many deaths among patients with bacteremia occur following hospital discharge.^34^ The methods used for ESBL testing have changed over time, as have data collection practices, and data were not always available on antibiotic use before blood draw, treatment regimen after diagnosis/culture, length of stay, other indicators of disease severity, and patient outcome after treatment. Our incidence rate calculations are underestimated, as our data only cover hospitalized patients. Exclusion of cases that grew a contaminant in the same hemoculture bottle as a pathogen led to slight underestimation (overall ESBL-producing *E. coli* incidence differed by 1.4% [42.1 versus 41.5 per 100,000 population] and *K. pneumonia* differed by 0.3% [13.6 versus 13.3]).

Our study also had several important strengths. The inclusion of smaller district-level hospitals and all laboratories with hemoculture capability helped provide a comprehensive estimate of ESBL incidence at the community level, and the use of automated hemoculture systems enhanced the sensitivity of organism detection.^22^ Furthermore, the use of this comprehensive data source allowed us to identify repeat cultures on the same patient and describe recurrent infections. Rigorous efforts were also made to identify and remove duplicate isolates to avoid multiple counting; failure to do this has been shown to overestimate incidence and resistance rates.^43^ Population-based studies such as this are considered the gold standard for accurately estimating the incidence of BSIs and other infectious diseases and comparing incidence rates over time and place.^44^ However, such studies are vanishingly rare in most parts of the world. A 2012 review of community-acquired BSIs did not identify any truly population-based incidence estimates from South or Southeast Asia, although one study extrapolated an incidence rate from an estimated capture rate for the catchment population.^45^ Data collected from other studies can result in biased estimates which frequently underestimate the total burden of disease but overestimate the frequency of antimicrobial resistance.^43,44^

Common issues leading to increased rates of drug resistance in many bacteria are the use of antibiotics without proper examination and diagnostic testing, for conditions not requiring antibiotics, the extensive use of antibiotics in the animal sector, and the use of overly broad-spectrum antibiotics. Surveillance systems are vital to monitoring emergence and trends in antimicrobial resistance. They are helpful for the development of rational therapy recommendations and policy for limiting the use of certain classes of antibiotics, especially in outpatient settings, and for enhancing efforts to prevent hospital-acquired infections which are more likely to be antibiotic resistant. These policies are especially important as an aging population will put more and more individuals in Thailand at risk of contracting difficult or impossible to treat bacterial infections. Laboratory-enhanced surveillance in Thailand is vitally important, given the high incidence of invasive ESBL-producing *E. coli* and *K. pneumoniae* bacteremia, together with the predicted increases in case counts for persons 50 years and older, and the emergence of carbapenemase resistance.
